# Speeding Up the Identification of Cystic Fibrosis Transmembrane Conductance Regulator-Targeted Drugs: An Approach Based on Bioinformatics Strategies and Surface Plasmon Resonance

**DOI:** 10.3390/molecules23010120

**Published:** 2018-01-08

**Authors:** Marco Rusnati, Davide Sala, Alessandro Orro, Antonella Bugatti, Gabriele Trombetti, Elena Cichero, Chiara Urbinati, Margherita Di Somma, Enrico Millo, Luis J. V. Galietta, Luciano Milanesi, Paola Fossa, Pasqualina D’Ursi

**Affiliations:** 1 Department of Molecular and Translational Medicine, University of Brescia, 25123 Brescia, Italy; marco.rusnati@unibs.it (M.R.); antonella.bugatti@unibs.it (A.B.); chiara.urbinati@unibs.it (C.U.); m.disomma@unibs.it (M.D.S.); 2 Institute for Biomedical Technologies, National Research Council (ITB-CNR), 20090 Segrate, Italy; d.sala1388@gmail.com (D.S.); alessandro.orro@itb.cnr.it (A.O.); gabriele.trombetti@gmail.com (G.T.); luciano.milanesi@itb.cnr.it (L.M.); 3 Department of Pharmacy, Section of Medicinal Chemistry, School of Medical and Pharmaceutical Sciences, University of Genoa, 16132 Genoa, Italy; cichero@difar.unige.it; 4 Department of Experimental Medicine, Section of Biochemistry, University of Genoa, 16132 Genoa, Italy; enrico.millo@unige.it; 5 Center of Excellence for Biomedical Research (CEBR), University of Genoa, 16132 Genoa, Italy; 6 Istituto Giannina Gaslini, 16147 Genoa, Italy; l.galietta@tigem.it

**Keywords:** cystic fibrosis, computational chemistry, molecular dynamics, molecular modeling, surface plasmon resonance

## Abstract

Cystic fibrosis (CF) is mainly caused by the deletion of Phe 508 (ΔF508) in the cystic fibrosis transmembrane conductance regulator (CFTR) protein that is thus withheld in the endoplasmic reticulum and rapidly degraded by the ubiquitin/proteasome system. New drugs able to rescue ΔF508-CFTR trafficking are eagerly awaited. An integrated bioinformatics and surface plasmon resonance (SPR) approach was here applied to investigate the rescue mechanism(s) of a series of CFTR-ligands including VX809, VX770 and some aminoarylthiazole derivatives (AAT). Computational studies tentatively identified a large binding pocket in the ΔF508-CFTR nucleotide binding domain-1 (NBD1) and predicted all the tested compounds to bind to three sub-regions of this main pocket. Noticeably, the known CFTR chaperone keratin-8 (K8) seems to interact with some residues located in one of these sub-pockets, potentially interfering with the binding of some ligands. SPR results corroborated all these computational findings. Moreover, for all the considered ligands, a statistically significant correlation was determined between their binding capability to ΔF508-NBD1 measured by SPR and the pockets availability measured by computational studies. Taken together, these results demonstrate a strong agreement between the in silico prediction and the SPR-generated binding data, suggesting a path to speed up the identification of new drugs for the treatment of cystic fibrosis.

## 1. Introduction

Cystic Fibrosis (CF) is the most common lethal monogenic disorder in Caucasians. It is due to different mutations in the cystic fibrosis transmembrane conductance regulator (CFTR), a protein composed of five domains: two nucleotide binding domains (NBD1 and 2), two transmembrane domains (MSD1 and 2) and one regulatory domain (R) [1]. The mutations causing CF are classified in five groups, according to the mechanism of CFTR loss-of-function [2]. The most common one, occurring in 70–90% of CF patients, is the deletion of phenylalanine 508 (ΔF508) in NBD1 that causes inappropriate folding and structural instability of ΔF508-CFTR that, for this reasons, remains trapped in the endoplasmic reticulum and is rapidly degraded by the ubiquitin/proteasome system.

Several molecular chaperones and co-chaperones, among which Hsp70/Hsc70, Hsp90, CHIP and BAG-2 [3] are involved in CFTR intracellular processing. In particular, keratin 8 (K8) plays an important role in ΔF508-CTFR retention and degradation, through a preferential binding to ΔF508-CTFR NBD1 with respect to its wild type (WT) counterpart. Accordingly, the inhibition of ΔF508-CFTR/K8 interaction leads to the rescue of the defective ΔF508-CFTR processing [4,5]. However, also if the mutant protein is targeted to the plasma membrane through rescue maneuvers, the probability that its channel is open is reduced. The deficit of CFTR at the plasma membrane has a major impact on the respiratory system, since CF patients produce a thick mucus that cannot be cleared, resulting in an impairment of innate defense against bacteria [6]. The resulting pulmonary infections cause a progressive loss of respiratory function that, in the last stages of the disease, may require lung transplantation as a life-saving intervention [7,8].

Current therapies are mostly aimed at treating CF symptomatically, e.g., by aggressive antibiotic strategies for the clinical management of bacterial lung infections. Although these therapies has significantly pushed forward the mean survival age of patients from early childhood in the 1950s to the late 30s at present [9], the burden of CF care remains very high and life quality and expectancy for most CF patients are still limited.

An ambitious therapeutic alternative is to address CF systemically, by means of small molecules that restore the trafficking (correctors) and gating (potentiators) capacity of mutated CFTR. At this regard, a big effort has been made in the last ten years to determine the complete human CFTR 3D structure by means of homology modelling, NMR and X-rays studies. In addition, computational studies and cell- or tissue-based assays have been used to elucidate the mechanisms of action of CFTR drugs, in particular VX809 [10,11,12,13,14,15].

In this context, the mutated CFTR protein and its NBD1 domain represent ideal targets for small molecules with rescuing activity on ΔF508-CFTR [10,16]. Leveraging high-throughput screening by companies and academic labs, this approach has already lead to some interesting compounds that however remain in need of better elucidation of their molecular mechanism of action. To this lack of knowledge surely contributes the fact that up to now, the available experimental structures of human WT or mutated NBD1 and complete CFTR are largely incomplete: as concerns NBD1, the human protein lacks some regions and/or contains solubilizing mutations (reviewed by Lewis and co-workers [17]). As for the recently released human CFTR model, its experimental structure presents unsolved portions [14], so that a detailed picture of the conformational changes which result in channel opening/closing remain to be fully resolved [18].

To clarify these points, possibly allowing a rational design and full characterization of potential CF drugs, here we exploited a synergistic approach including high performance computational studies of the murine NBD1 protein (available as complete WT and ∆508F mutated structures) and biomolecular interaction analysis by surface plasmon resonance (SPR) for an accurate validation of the in silico predictions. The former allows the full evaluation of the dynamic behavior(s) of proteins, simulating real biological events such as the molecular interactions between a ligand and its target and the conformational changes of CFTR variants in respect to the WT protein. The latter represents the golden standard label-free technology for real-time analysis of molecular interactions also in the field of CF drug discovery [8,19], being able to generate high-quality data on affinity, kinetic and mechanistic aspects of interactions occurring between target proteins and drug candidates [20,21].

**Figure 1:**
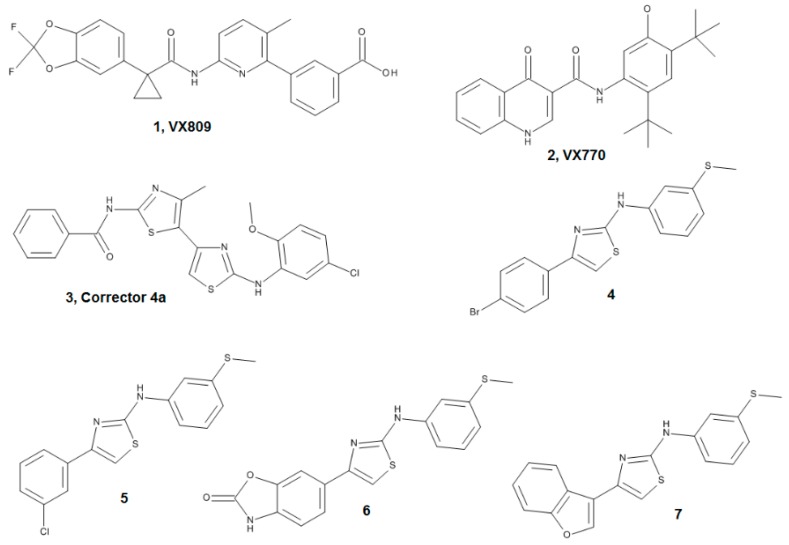
Structures of ligand dataset.

To solidly validate the complementary approach made up by computational and SPR studies, here we have investigated the interaction of three well characterized and promising CF correctors and four new potentially interesting compounds (Figure 1) [22,23,24,25,26] and characterized their ability to bind to ΔF508-NBD1.

The results obtained by the computational approach resulted in agreement with SPR findings, providing a detailed characterization of the ligand-protein interaction useful for an efficient screening of large compound libraries, a better rational design and a tailored synthesis of new lead compounds for the rescue of mutated CFTR.

## 2. Results

### 2.1. Characterization of the ΔF508-NBD1 Flexibility by Molecular Dynamics Simulations (MDS)

**Figure 2:**
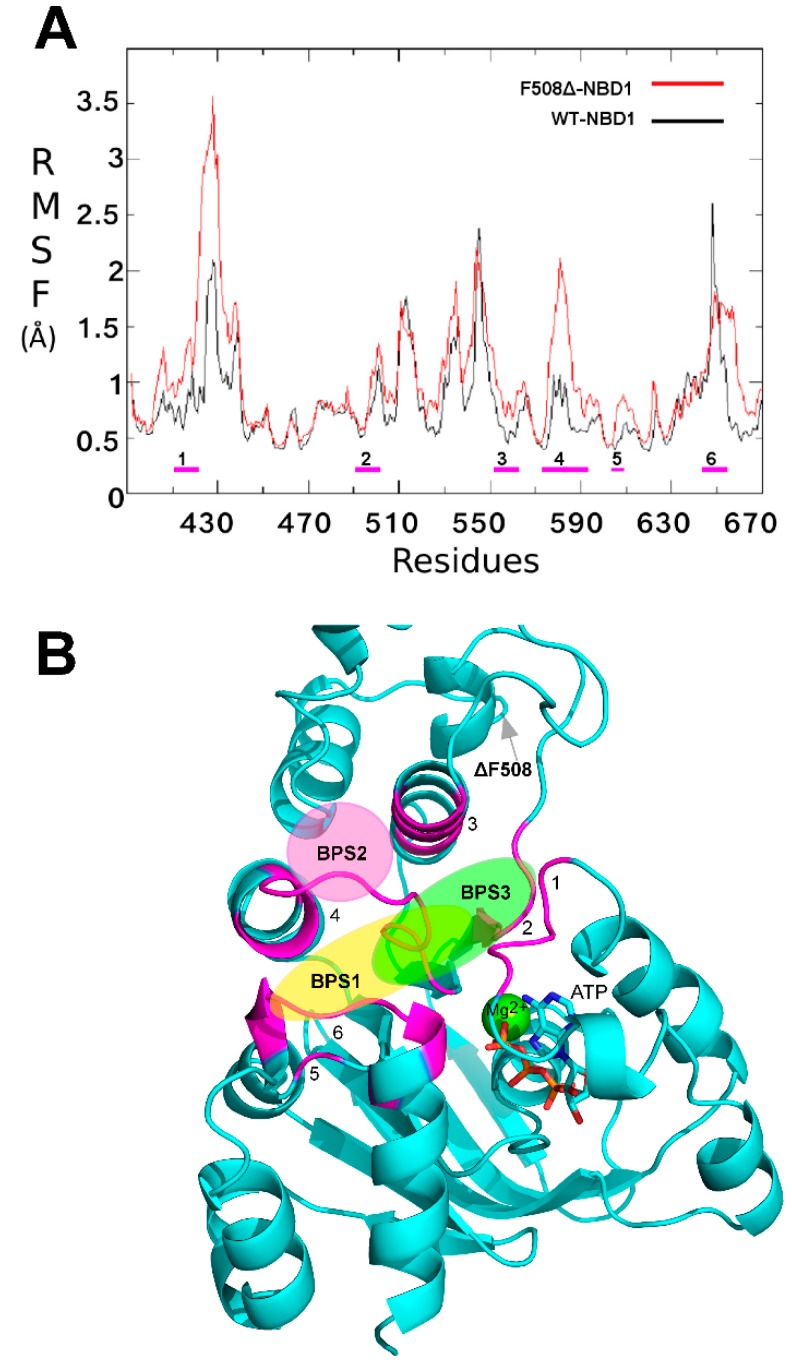
(**A**) Root mean square fluctuation (RMSF) of ΔF508-NBD1 protein. RMSF values for ΔF508 and WT NBD1 are reported in red and black respectively. Magenta bold dashes indicate the regions composing the main binding pocket, numbered from one to six; (**B**) Ribbon representation of the ΔF508-NBD1 protein. The regions forming the main binding pocket predicted by CASTp are highlighted in magenta and numbered from one to six. The three binding sub-pockets predicted for the data-set ligands are also indicated as colored circles: yellow, binding sub-pocket 1 (BSP1), the putative binding site for VX809; magenta, BSP2, the putative binding site for compound **5**; light green, BSP3, the putative binding site for compounds **4** and **6**. The grey arrow indicates the location of the ΔF508 mutated residue.

Several studies demonstrated that the ΔF508-NBD1 apo is thermodynamically and kinetically destabilized at physiological temperature in respect to the WT NBD1 apo [27,28,29]. In order to highlight which differences in structure and backbone flexibility were induced in the protein by the mutation, we firstly focused our attention on exhaustive MDS studies of 200 ns on both WT NBD1 and ΔF508-NBD1 forms. In agreement with literature data [30,31] a very high flexibility was observed for ΔF508-NBD1 in comparison to the WT NBD1, that was not limited to the close environment of the ΔF508 residue (Figure 2A).

To compare the flexibility of ΔF508 and WT NBD1, we computed the root mean square fluctuation (RMSF), an average measure of atomic mobility of backbone atoms along the MDS. Results show very high flexibility (Figure 2A) in five loops contained in a large region that forms the binding pocket (predicted by CASTp program) and that includes residues from a highly conserved regions in all NDB proteins, such as the Walker B motif, the signature sequence LSGGQ, the Q- and the H-loops (Figure 2B) [32].

### 2.2. Binding Sites Prediction

**Figure 3:**
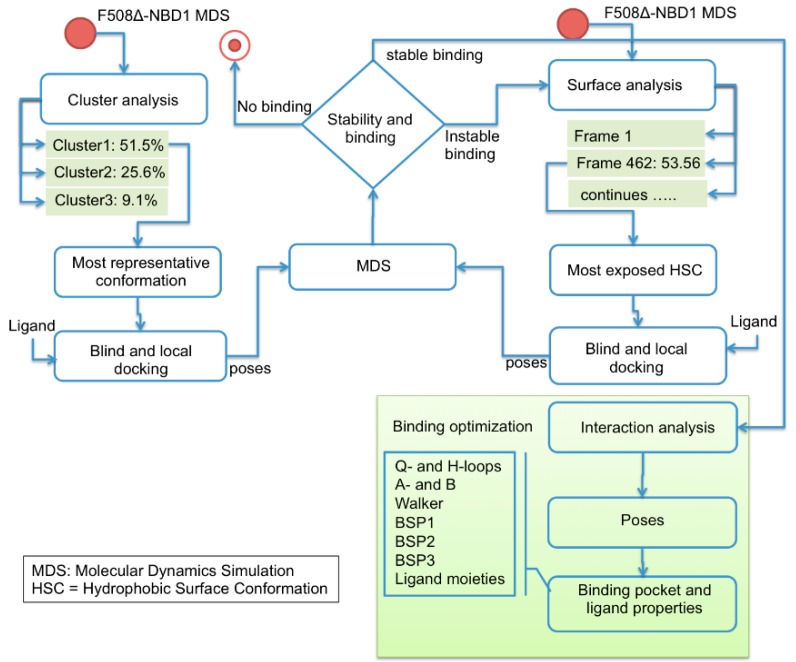
Workflow of the computational approach applied for binding site prediction and optimization.

It is expected that conformational changes within NBD1 impact its molecular interaction with small molecules. Thus, cluster analysis was exploited to select from MDS trajectory the most representative ΔF508-NBD1 conformation. Three clusters were obtained (with 51.5%, 25.6% and 9.1% of frames grouped with a distance <2 Å). From the most populated one we extracted the most representative conformation that was then used to perform docking analysis with the ligands (Figure 3) using both blind and local approaches. As a result, a putative binding site and binding mode could be rationally inferred for each ligand. For aminoarylthiazole (AAT) derivatives, equi-populated clusters of solutions with similar energy and inverse orientation of the ligand were observed in each run (Supplementary Materials, Table S1, Figures S1–S3).

**Figure 4:**
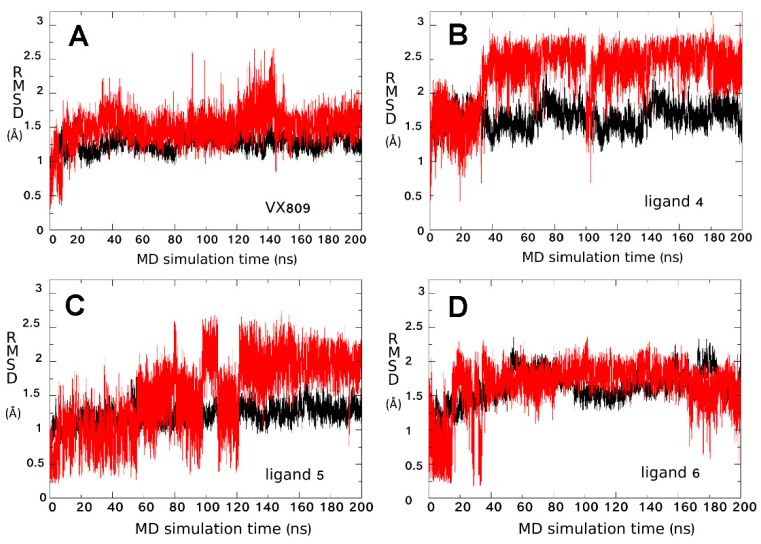
Analysis of putative binding mode stability. RMSD along simulation time for the ΔF508-NBD1/ligand complexes calculated with respect to the most representative conformation of the ΔF508-NBD1/ligand trajectory obtained from cluster analysis. The ligand and the ΔF508-NBD1 RMSD are depicted in red and in black, respectively. (**A**) VX809; (**B**) ligand **4**; (**C**) ligand **5**; (**D**) ligand **6**.

Additional MDS were performed for each complex obtained from the docking calculations to optimize the orientation and pose of the ligand in the binding site and to highlight the ligand’s moieties involved in its interaction with the NBD1 domain. In addition, the values of root mean square deviation (RMSD) between the starting and the terminal positions during MD simulations were calculated for each ligand to evaluate its stability inside the BSP. VX809 and the two AAT derivatives **4** and **6** retain their initial position consistently during MDS, thus remaining steadily anchored to ΔF508-NBD1 via the same interactions all along the trajectory (Figure 4).

A highly unstable binding was instead observed during the simulation for AAT derivative **5** (Figure 4C and further investigated below), for the inactive AAT derivative **7** (included in the study to test the validity of the experimental protocol), for corrector 4a, (C4a) [11] and for VX770 (included in the study since they have been proposed to act by a mechanism of action independent from the NBD1 module) (data not shown). Accordingly, SPR analysis demonstrated that C4a and VX770 actually do not bind to ΔF508-NBD1 (Table 1).

The binding instability of compound **5** observed during MDS (Figure 4C) was further investigated in order to predict the protein conformation able to give a stable ligand-protein complex. Analysis of the RMSD fluctuations as a function of time for the complex showed instability reflecting an unfolding of the C/N terminal of the helix 9 X-ray structure.

**Figure 5:**
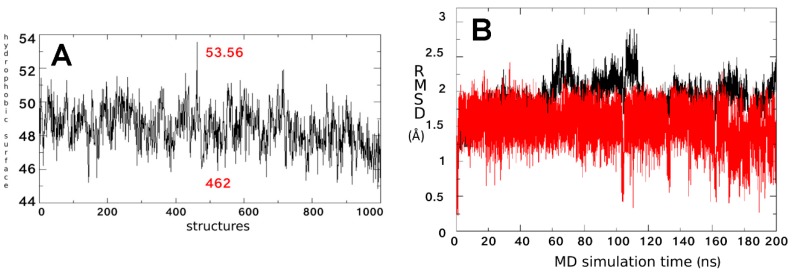
(**A**) Fluctuation of the hydrophobic surface area of ΔF508-NBD1 calculated considering 1000 equidistant structures along its dynamics (one each 200 ps). The frame number 462 with the most exposed hydrophobic surface used for compound **5** docking studies and 53.56, the value corresponding to its hydrophobic surface in Å^2^, are indicated in red; (**B**) RMSD of atomic loci along the MDS trajectory of the ΔF508-NBD1/compound **5** complex calculated in respect to the frame number 462 and plotted against simulation time. Black and red lines refer to ΔF508-NBD1 backbone and ligands, respectively. All structure indices in x-axis must be multiplied by 1000 to obtain the frame index.

A broader conformational freedom of ΔF508-NBD1 apo in respect to that of the WT NBD1 apo was observed, mainly due to the reorganization of the interactions network between residues and causing the exposure of a wider hydrophobic region [31]. On this basis, we searched for the best ΔF508-NBD1 conformation able to bind compound **5** in a stable way: by performing a hydrophobic surface analysis considering 1000 equidistant frames along the MDS of the ΔF508-NBD1 apo (one each 200 ps), we observed that the hydrophobic exposed surface spanned from 45 to 53 Å^2^ (Figure 5A).

The frame with the most hydrophobic exposed surface was then taken in consideration as the potential protein conformation able to stably bind compound **5** and used to repeat docking studies (Figure 3, right side). In this way, a new binding site could be predicted where the ligand locates with high stability, at variance with what observed for the binding site previously predicted with the docking simulations based on the most representative conformation of ΔF508-NBD1 apo (Figure 4C).

### 2.3. Ligand Interactions on ΔF508-NBD1

**Figure 6:**
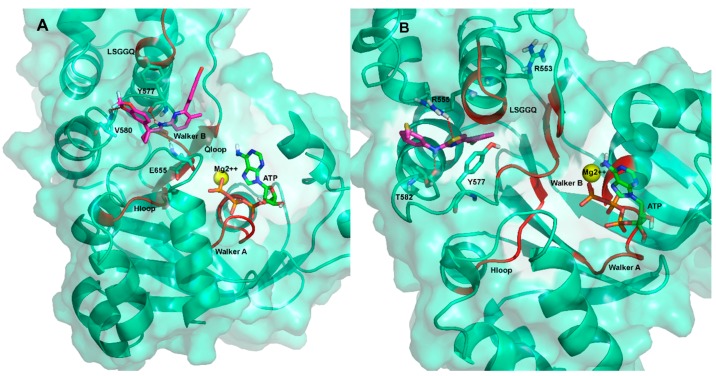
Predicted binding mode of VX809 (**A**) and compound **5** (**B**) with ΔF508-NBD1. The residues involved in the H-bonds between the ligands and the mutated protein are shown as sticks in red dotted line. The protein is shown as ribbon. Conserved NBD signature sequences are shown as red ribbon.

Ligand interactions information obtained from MDS predicted that the most effective ligand VX809 is anchored in the BSP1 region of ΔF508-NBD1 by three H-bonds, the first between its amidic nitrogen and Y577, the second between its carbonyl group and E655 and the third between one of its catechol oxygens and V580 (Figure 6A and Supplementary Materials, Figures S4 and S6).

Binding of compound **5** seems to occur in another NBD1 sub-pocket (BSP2) via a network of H-bonds: one between the sulphur atom of the AAT ring and R555, a very important residue of the RXR motif [33], another between the hydrogen of the aminic nitrogen of compound **5** and T582. Also, a π-π interaction between the thiazolic ring of the AAT and Y577 seems to occur that further stabilizes the interaction (Figure 6B and Supplementary Materials, Figure S6).

**Figure 7:**
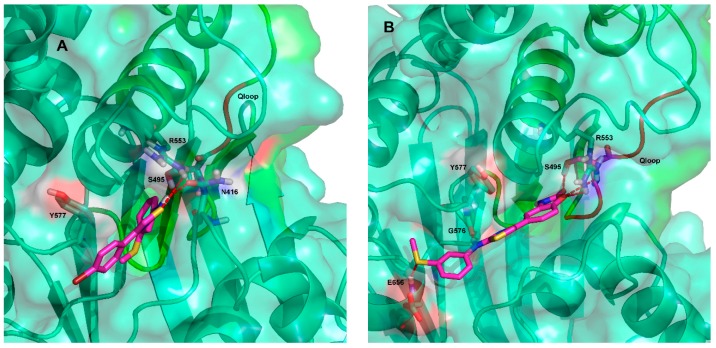
Predicted binding mode of compound **4** (**A**) and compound **6** (**B**) with ΔF508-NBD1. The residues involved in H-bonding interactions with the ligands are shown in sticks. The protein is shown as ribbon and Connolly surface. The binding region of K8 suggested by Odolczyk [34] is shown in green.

Compounds **4** and **6** seem to bind to a further different sub-pocket (BSP3). Compound **4** making two H-bonds between the sulphur atom of its thiazole ring and R553 and between its amidic nitrogen and N416 (Figure 7A and Supplementary Materials, Figure S5). Concerning the compound **6**/ΔF508-NBD1 interaction, cluster analysis on MDS trajectory predicted three equipopulated clusters with a 17% of occurrence each. This result is different from the other complexes analyzed, where occurrences of the most representative cluster are 46%, 38% and 33% for VX809, compound **4** and compound **5**, respectively. In all the clusters, a H-bond between compound **6** and S495 (a residue located in the Q-loop) can be predicted, while only in one cluster a transient H-bond between compound **6** and R555 seems to occur, due to alternative orientations of the side chain of the residue during the dynamics. Thus, at variance with VX809, all the other compounds studied seem to bind ΔF508-NBD1 via residues belonging to the conserved NBD signature sequences, further supporting the hypothesis of different binding modes of the ligands to NBD1. Important to note, the binding modes here proposed for VX809 do not fully agree with some literature results proposing binding poses at the ICL4:NBD1 inter-domain interface and along strand S9 and strand S10 [10,11,12,13,15]. It must be pointed out however that all these studies were performed on RI-del human NBD1, without performing MDS to evaluate the stability complex.

**Figure 8:**
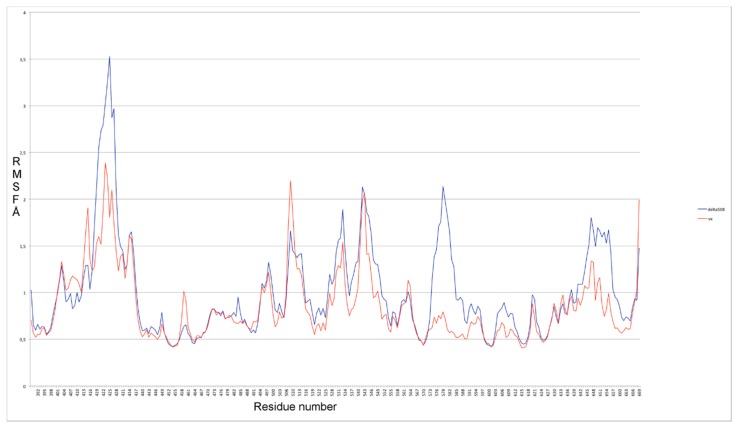
RMSF profiles obtained from VX809 binding pose in comparison with ∆F508-NBD1.

In addition, the RMSF profiles obtained from our VX809 binding pose, selected by docking and followed by MDS, show an attenuation of the fluctuation along almost all regions in ∆F508-NBD1 when compared with the WT protein (Figure 8). This result is in agreement with literature data reporting reduced fluctuations induced by VX809 in 499-502 and 539-542 regions near ICL3 of ∆F508-NBD1 [35].

Some CFTR-targeted drugs exert their action by impairing the binding of ΔF508-NBD1 to K8, thus escaping the ubiquitin-proteasome machinery. This observation, together with the different positioning of the various AAT derivatives in the NBD1 module, prompted us to evaluate the capacity of the various compounds studied to interfere with the K8/ΔF508-NBD1 interaction. As a result, the binding pockets of VX809 and **5**, as determined by MDS and docking (BSP1 and BSP2, respectively), turned out to be distinct from the binding site of K8 already reported by Odolczyk [34]. The binding modes of compounds **4** and **6** indicated instead that they share part of their binding pocket with the K8 anchoring cleft. In details, the aromatic ring of compound **4** bringing the methylsulphur moiety seems to be partially inserted into the anchoring cleft of K8 (Figure 7A), while the 2-oxazolidinone moiety of compound **6** appears to be completely inserted into an inner portion of the K8 anchoring cleft, with the remaining part of the molecule situated in the adjacent sub-pocket, making two H-bonds between its aminic nitrogen and G756 and between the sulphur atom of its methylsulphur moiety and E656 (Figure 7B and Supplementary Materials, Figure S7).

### 2.4. Interaction Analysis by Surface Plasmon Resonance

**Figure 9:**
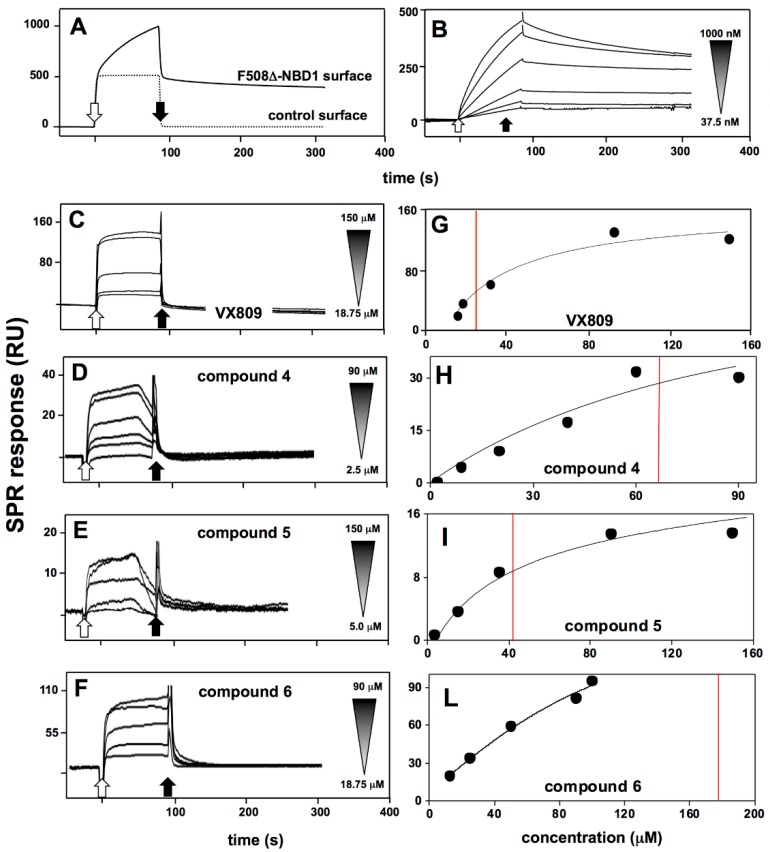
SPR analysis of the interaction of ΔF508-NBD1 with K8 or with the putative CF drugs. (**A**) Sensorgrams showing the binding of K8 (600 nM) to a ΔF508-NBD1- or to a control BSA-coated surface; (**B**) Blank-subtracted sensorgrams overlay showing the binding of increasing concentrations of K8 (1000, 600, 300, 150, 75, 37.5 nM) to the ΔF508-NBD1-coated sensor chip; (**C**–**F**) Representative blank-subtracted sensorgrams overlays showing the binding of increasing concentrations of the indicated compounds to the ΔF508-NBD1-coated sensor chip. In all the panels, response (in RU) was recorded as a function of time. White and black arrows mark the beginning and the end of the injection of K8 or of the compounds; (**G**–**L**) Steady-state analysis obtained by fitting the proper form of Scatchard’s equation for the plot of the bound RU at equilibrium versus the ligand concentration in solution for the indicated compounds injected onto the sensor chip-immobilized ΔF508-NBD1. Red lines indicate the calculated (**G**,**H**) or presumed (**L**) *K_d_* values.

SPR analyses were finally performed to validate the in silico predictions that, as already stated above, is the main aim of this work. In a first series of experiments, to check the correct immobilization and binding availability of ΔF508-NBD1 onto the sensor chip, we performed binding analyses with K8 in those same experimental conditions used to analyze the binding of the putative CF drugs (see Materials and Methods). As shown in Figure 9A, when tested at 600 nM, K8 specifically binds to immobilized ΔF508-NBD1 but not to BSA surface (here used as a negative control). When increasing concentrations of K8 were injected onto the ΔF508-NBD1 surface, the blank subtracted sensorgrams overlay shown in Figure 9B was obtained: K8/ΔF508-NBD1 interaction occurs in a saturable and very stable way, being K8 detachment from the sensor chip after the end of the injection very slow.

Once validated the experimental model, the direct interaction of the putative CF drugs with ΔF508-NBD1 was investigated. In agreement with computational predictions, VX770, C4a and compound **7** do not bind to ΔF508-NBD1 (Table 1). The other compounds bind to ΔF508-NBD1 but, at variance with K8, their interaction is characterized by a very quick detachment from the sensor chip after the end of the injection (Figure 9A–D). For compounds **4** and **5** in particular, their premature detachment from surface-immobilized ΔF508-NBD1 can be appreciated even during the association phase, causing an anomalous shaped sensorgram. This may be explained by the limited solubility of small chemical compounds and their tendency to aggregate during the SPR analysis also in the presence of DMSO [36].

Blank subtracted values of RU bound at equilibrium obtained from injections of increasing concentrations of the various putative CF drugs on ΔF508-NBD1 were then used to build the curves reported in Figure 9E–H: VX809, compounds **5** and **4** bind to ΔF508-NBD1 in a dose-dependent and saturable manner, with *K_d_* values ranging between 24.2 ± 6.2 and 99.3 ± 14.5 μM. At the concentrations tested, compound **6** shows no saturable binding, with a presumed *K_d_* value equal to 197.9 ± 4.5 μM (Table 1). 

In silico studies predicted VX809 and compound **5** to locate in two distinct regions of ΔF508-NBD1 (BSP1 and 2, respectively) and compounds **4** and **6** to locate in region BSP3 proposed to be also involved in the binding to K8 [37]. These observations prompted us to evaluate the capacity of the various compounds to affect the binding of K8 to ΔF508-NBD1. In a first series of experiments, exploiting the slow dissociation rate of the K8/ΔF508-NBD1 interaction, a saturating amount of the chaperone was injected over the ΔF508-NBD1 surface and allowed to reach an equilibrium binding. Subsequently, the putative drugs were injected.

**Figure 10:**
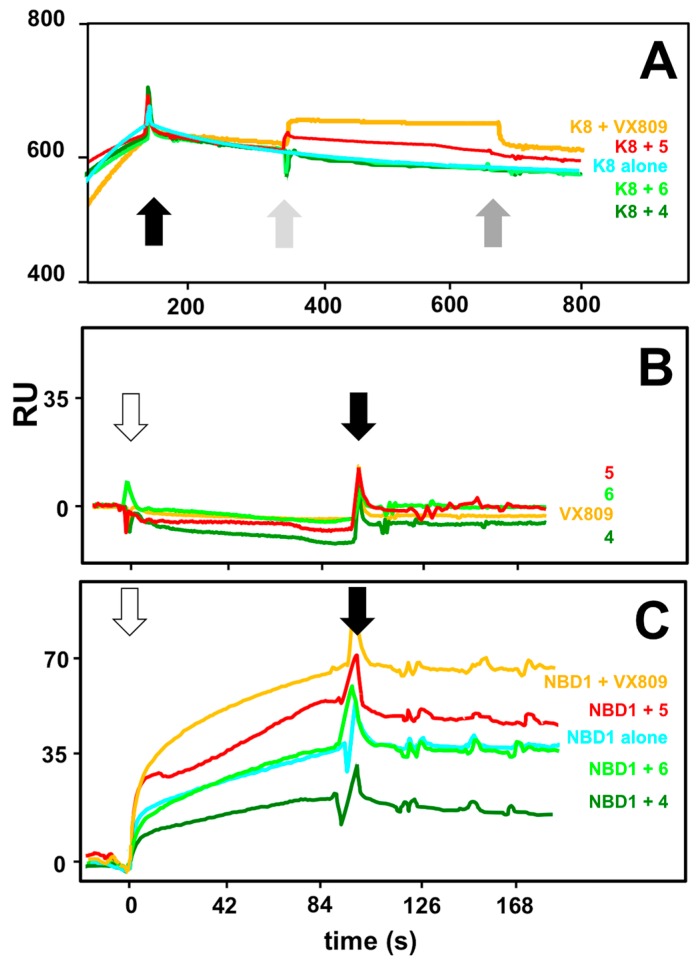
Effect of the putative CF drugs on the K8/ΔF508-NBD1 interaction. (**A**) Representative sensorgrams showing the injection of the various compounds (at 150 μM) onto ΔF508-NBD1-coated sensor chip saturated with K8. The black arrow marks the end of K8 injection. Light and dark grey arrows mark instead the beginning and the end of the subsequent injection of the various compounds on the K8-saturated surface; (**B**) Representative sensorgrams showing the injection of the various compounds (500 μM) onto K8-coated sensor chip. White and black arrows mark the beginning and the end of the injection of the compounds, respectively; (**C**) Representative sensorgrams showing the injection of ΔF508-NBD1 (600 nM) onto K8-coated sensor chip in the absence or in the presence of the indicated compounds (500 μM). White and black arrows mark the beginning and the end of the injection of the various mixtures, respectively. In all the panels, the response (in RU) was recorded as a function of time. The sensorgrams shown are representative of other two that gave similar results.

As shown in Figure 10A, injection of VX809 or of compound **5** on the K8-saturated ΔF508-NBD1 surface results in an increase of bound RU, indicating that these two compounds retain the capacity to bind to ΔF508-NBD1 also when the protein is already engaged by K8. Relevant to this interpretation, none of the compounds studied is able to bind to K8 (Figure 10B). The inability of compounds **4** and **6** to alter the binding of K8 to ΔF508-NBD1 (Figure 10A) may be due to the fact that the ΔF508-NBD1/K8 interaction is too stable to be disrupted by the subsequent injection of the two compounds that indeed turned out to be the two weakest ΔF508-NBD1 binders (Table 1). In any case, the capacity of both the AATs and K8 to bind to immobilized ΔF508-NBD1 makes this experimental model inappropriate to study possible competitive effects. To overcome this limit, we reversed the SPR model by immobilizing K8 and evaluating its capacity to bind ΔF508-NBD1 (injected at 600 nM) in the presence of the compounds at a dose (500 μM) compatible with the highest concentration of DMSO (5%) that does not hamper the K8/ΔF508-NBD1 interaction (data not shown). As already mentioned, none of the compounds interacts with immobilized K8 in these experimental conditions (Figure 10B). As shown in Figure 10C, when injected alone, ΔF508-NBD1 binds to immobilized-K8 (35 RU at equilibrium). The injection of ΔF508-NBD1 in the presence of VX809 or compound **5** results in an interaction that is quantitatively higher (68 and 45 RU at equilibrium, respectively), confirming the results obtained in the previous SPR assay. On the contrary, in the same experimental conditions, compound **4** exerts a 50% inhibition of the ΔF508-NBD1/K8 interaction, indicating that it competes with immobilized K8 for the binding to ΔF508-NBD1 while compound **6** resulted again ineffective, possibly because of its extremely low affinity interaction with ΔF508-NBD1 (Table 1). In conclusion, also when dealing with the effect of the drugs on ΔF508-NBD1/K8 interaction, computational predictions are validated by experimental SPR analysis.

A last relevant aspect is the statistically significant correlation that exists, for each compound, between the values of maximal RU bound to sensor chip-immobilized ΔF508-NBD1 at equilibrium and the different time availability of the ΔF508-NBD1 pockets as calculated from the docking poses simulation along the MD of the protein in apo form (Supplementary Materials, Figure S4). VX809 binds to the highest number of available ΔF508-NBD1 conformations (48%) and, accordingly, it shows also the highest binding capacity when tested in SPR (Supplementary Materials, Table S2). The AAT derivatives behave differently, with compounds **5** and **4** able to bind only 15% of the available conformations and compound **6** showing an intermediate capacity (22%).

Again, these values quantitatively correlate to the values of maximal RU bound at equilibrium (Supplementary Materials, Table S2). The disclosed correlation between these latter values and the different time availability of the ΔF508-NBD1 pockets suggests that ΔF508-NBD1 retains its capacity to present different conformations also when immobilized to the sensor chip. As a result, the different time availability of its distinct binding pockets reflects in a different binding capacity that could be not directly related to the measured binding affinity. The take-home message is that both pocket availability (as predicted by computational studies) and the values of maximal binding at equilibrium (as calculated by SPR analysis) should be taken into account when evaluating the efficacy of a potential lead compound, further underlying the validity of the double approach here proposed.

## 3. Discussion

In this work, by means of extensive MDS simulation and docking, we have studied the interaction of ΔF508-NBD1 with a series of AAT derivatives and VX809, selected as template. The results of these studies allowed the prediction of putative binding modes with statistics of occurrence that finally turned out to be in agreement with actual SPR binding analysis, linking solidly these two complementary techniques. This in-tandem approach, here applied to the murine NBD1 domain, can contribute to unravel the complex puzzle concerning the mechanism of action of small molecules in CFTR-targeted therapy, an issue that is commonly acknowledged as not fully understood, due mainly to the still present limits of the experimental structures of human NBD1 and complete CFTR (see Introduction).

The computational investigations on ΔF508-NBD1 here performed provided novel insights regarding the binding mode of known and novel potential CF drugs: the well-known VX809 was predicted to strongly anchor to the central cleft of NBD1 via three H-bonds. Accordingly, when compared to the ATT derivatives in SPR analysis, it shows the highest binding affinity for ΔF508-NBD1 (Table 1). In respect to VX809, the three AAT derivatives **4**, **5** and **6** seem not to share the same binding site, even if endowed of the same chemical scaffold. In details, compound **6** appears to occupy a sub-region inside the main pocket, orienting its carbonyl group towards S495 in the Q-loop. Compound **4** presents a slightly different predicted binding since in its scaffold is present a *p*-bromophenyl moiety that is less polar than compound **6** and whose carbonyl group causes the molecule to accommodate in the same sub-pocket but in a different mode, flanking the RXR motif. Interestingly, modelling and mutagenesis studies on NBD1/NBD2 dimers showed that activator compounds strongly interact with the NBD1 domain in a site that includes part of the Q-loop, of the LSGGQ signature, of switch-II of NBD1 plus the Q-loop of NBD2 [38]. These computational findings support the evidence that compound **6** acts as a potentiator [26]. Furthermore, NMR spectra of various NBD1 mutants support the evidence that deletion of Phe-508 affects Q-loop conformation inhibiting dimerization, possibly explaining the mechanism by which the mutation impairs the opening of the channel [38] and rationally supporting the selection of ΔF508-NBD1 as the primary target for designing new activators. 

Compound **5** is predicted to bind to BSP2 inside the main pocket, closer to the LSSGQ conserved residues and specifically interacting with R555 of the RXR motif, a conserved portion of NBD1 already reported to be important for CFTR correct folding and stability [39]. Thus, a possible mechanism of action of compound **5** can be its capacity to prevent the abnormal accessibility of RXR in ΔF508-NBD1, thus preventing mistrafficking of the mutated protein. Also, the predicted different binding modes of compound **5** and VX809 are in agreement with the observation that the two compounds show a synergistic effect in vitro [26].

The predicted distinct binding modes of the AAT derivatives correlate to their different scaffold decorations and support their double biological activity [23]. In fact, according to our calculations, the interaction of compound **6** with S495 residue in the Q-loop may promote the dimerization of NBD1 with NBD2 and thus the following channel opening (potentiator activity). On the other hand, the predicted H-bonds of compounds **5** and **4** with the arginine residues of the RXR motif may alter the interaction between ΔF508-NBD1 and the proteostasis components (CFTR interactome) allowing the protein to reach the plasma membrane (corrector activity).

Finally, it is interesting to note that the binding modes we have inferred by MDS and docking suggest that compounds **4** and **6**, but not VX809 and compound **5**, partially insert in the proposed K8 binding site of ΔF508-NBD1, each one in a specific way.

These predicted binding modes were validated by SPR analysis that showed that compound **4** displaces K8 from its binding to ΔF508-NBD1, while VX809 and compound **5** can bind the protein simultaneously to K8. This also suggests the possibility that the well demonstrated rescuing capacity of VX809 in vivo [23,26] is K8-independent, being possibly mediated though the disruption of the interaction of ΔF508-NBD1 with chaperone(s) other than K8. This calls for further studies aimed at evaluating the effect of VX809 (as well as of other compounds) on chaperones different from K8 (i.e., Hsp70/Hsc70, Hsp90, CHIP and BAG-2) [3]. On the other hand, in silico predictions and SPR analysis point to compound **4** as a very interesting lead compound being able to displace 50% of K8 in its binding to ΔF508-NBD1. In effect, as already reported by Colas et al. [4], the disruption of K8/ΔF508-NBD1 interaction leads to the functional correction of the mutated protein. Further studies are required to fully clarify these points since the specific residues of ΔF508-NBD1 that directly interact with K8 (as well as with other chaperones) still need to be experimentally defined. 

In conclusion, the in-tandem approach made up of in silico studies and SPR analysis points to different binding modes (and different binding affinity) for VX809 and the various AATs that, in turn, indicate two potential rescue mechanisms: the first consisting in the capacity of the compound (i.e., VX809 and compound **5**) to stabilize the conformational variability of NBD1, the second consisting instead in the capacity of the compound (i.e., compound **4** and possibly compound **6**) to interfere with the binding of K8 to ΔF508-NBD1.

Finally, it is interesting to note that the SPR-generated values of RU bound at equilibrium to ΔF508-NBD1 and the docking poses of ΔF508-NBD1-ligand evaluated by inverse docking technique (Supplementary Materials, Table S2) correlate in a statistically significant way (Supplementary Materials, Figure S4), suggesting that the former parameter depends on the actual availability of the binding pockets that are variably exposed during the structural fluctuation of ΔF508-NBD1. In turn, this indicates that, once immobilized to a surface, the mutated protein maintains (at least in part) its capacity to move in the space, assuming those different conformations assessed by computational analysis.

The results of this work open up to further researches with multiple purposes: (a) to validate the putative binding modes of the compounds with ΔF508-NBD1 mutagenesis in those amino acids predicted to be involved in the interactions; (b) to correlate the various disclosed binding modes to different recovery effects of the compounds on ΔF508-CFTR by means of in vitro experiments; (c) to study the effect of selected compounds on the interaction of ΔF508-NBD1 with chaperones other than K8; (d) to speed up the design and synthesis of new and more efficient AAT derivatives, able to correct the ΔF508-NBD1 defect; (e) to better understand the molecular mechanisms of interactions and action of the new compounds, useful for a more exhaustive knowledge of CFTR disease.

## 4. Materials and Methods

### 4.1. Computational Studies

#### 4.1.1. Ligand Dataset

The ligand dataset has been chosen including three well known compounds provided by L.J.V. Galietta and here used as references: VX809 (Lumacaftor, one of the most effective correctors [24]); VX770 (Ivacaftor, a potentiator that does not bind to NBD1 [22] and that has been recently approved for CF therapy by both FDA and EMEA); C4a that, as VX770, does not bind to NBD1 [11,25]. Four new AAT derivatives, already synthesized by some of us, were also included: compounds **4**, **5**, **6** and the inactive compound **7** [23,26] (Figure 1). All compounds were built, parameterized (Gasteiger-Hückel method) and minimized with OMEGA 2.5.1.4: (OpenEye Scientific Software, Santa Fe, NM, USA, www.eyesopen.com) using MMFF94 force field. Regarding VX809 (characterized by a carboxylic moiety), accurate pKa predictions performed with ChemSpider (a free web tool from the Royal Society of Chemistry that can be found at: http://www.chemspider.com) let us to reasonably suppose that it maintains its anionic form at physiological pH, and calculations were performed accordingly.

#### 4.1.2. Model Refinement

Since the WT and ΔF508-NBD1 proteins available for our SPR analysis were the murine forms obtained from CFTR folding consortium (see below), we performed all computational studies using the murine crystallographic structures. 

WT- and ΔF508-NBD1 models (spanning residues 389–673) were based on the crystal structure of NBD1 from mouse CFTR, 1R0Z [40] and 3SI7 [41]. The phosphorylation on residues Ser422, Ser659, Ser660, and Ser670 were removed. Both structures presented an ATP-Mg^2+^ complex. The gap of six residues spanning positions 414–419 present on the WT NBD1 was modelled ab initio with loop refinement algorithm of Modeller [42], a tool relying on a scoring function and on an optimization schedule suitable for loop modelling. In details, hundreds of loop models were generated and their potential energy was evaluated with the DOPE scoring function. The lowest energy loop model was then chosen and the resulting WT model was used as a template to bridge the gap in the mutant domain (spanning residues 414–428).

#### 4.1.3. MDS Simulations

MD simulations were carried out using Amber 12 Molecular Dynamics Package. The compounds and the proteins were parameterized by means of the empirical charge model AM1-BCC (using the Antechamber module) and of the AMBER99SB-ILDN force field, respectively. The NBD1 module of CFTR was capped with an acetyl group on its N-terminus and with a *N*-methylamide on its C-terminus. The complex was solvated in a truncated octahedron box (8 Å distance between protein and box wall) using the explicit TIP3P water model. Na^+^ ions were added to neutralize the whole system. Minimization of the system was performed by several stages of steepest descent and conjugate gradient to remove bad contacts and release restraints. 

Using NVT conditions the system was weakly coupled to a temperature bath of 300 K under control with the Langevin algorithm that uses a simple Leapfrog integrator to propagate the dynamics. Next, in NPT conditions, the Berendsen pressure coupling of 1 atm with a collision frequency of 2 ps^−1^ was applied. After equilibration, MDS trajectories were carried out for 200 ns with an integration step of 2 fs. All the bonds involving hydrogen atoms were constrained by the SHAKE algorithm. The non-bonded cut-off distance was set to 8 Å and long range electrostatic interactions were calculated using Particle Mesh Ewald (PME) method.

#### 4.1.4. Docking Studies, Ligand-Protein Docking

The most representative conformations were obtained from the most populated cluster of the ΔF508-NBD1 MDS using the backbone atoms to compute the distance between each pair of frames (Figure 3, left side). A sampling of 10,000 frames along the trajectory was used for cluster analysis. This conformation was used to perform ligand-protein docking calculations. We checked that the docking poses were correctly inside the docking binding sub-pockets in the putative binding pocket predicted by CASTp [43]. Autodock Tools [44] was used to prepare proteins and ligands for the docking computations and to analyze the results. Polar hydrogen atoms were added to proteins and ligands and non-polar hydrogen atoms were merged. Finally, Gasteiger charges were added to all atoms. Protein coordinates were set to be rigid while ligand bonds were set to be rotatable. Blind (no specific binding pocket is searched) and local (docking targeting a specific binding pocket) protocols were performed to predict the binding sites for each ligand of the dataset. In particular, in blind docking calculations, a grid box encompassing the entire NBD1 structure was generated to take in consideration all the possible NBD1 binding sites. Calculations were carried out using Auto-Dock Vina [45]. The exhaustiveness of the global search was set to 64. The complex models with the lowest energy (based on an evaluation of AutoDock Vina’s empirical scoring function) were selected.

A local docking set of experiments was performed to predict the binding of each ligand of the dataset and to optimize the ligand-protein interactions. Both Autodock Vina and AutoDock4 were used for local docking and finally only the latter was used due to ambiguities (same energy and different pose) in the choice of the best pose in Autodock Vina results. AutoDock4 was used with a grid box centered on the binding regions predicted in the blind docking calculations. The affinity maps for all the atom types present, as well as an electrostatic map, were computed with a grid spacing of 0.375 Å. The search was carried out with the Lamarckian Genetic Algorithm, with the number of generations, energy evaluations and docking runs set to 27,000, 2,500,000, and 100 respectively [44]. Considering only the most populated clusters, we retained a set of two or three clusters with similar energy and numerosity. For each cluster we selected the pose with the lowest energy and used the corresponding complex to run an MD simulation for 200 ns using the PME method. In this way we filtered out the dynamically unstable poses retaining only those that were stable.

This procedure allowed us to predict a stable docked pose for VX809, compounds **4** and **6** but not for compound **5** for which we used another approach: hydrophobic surface analysis performed on 1000 frames along the MDS trajectory of ΔF508-NBD1 allowed the prediction of the frame with the most exposed hydrophobic surface (Figure 3, right side). Thereafter, the procedure was identical to the one described above (blind docking, MDS and stability analysis).

#### 4.1.5. Docking Studies, Inverse Docking Technique to Calculate Binding Propensity

**Figure 11:**
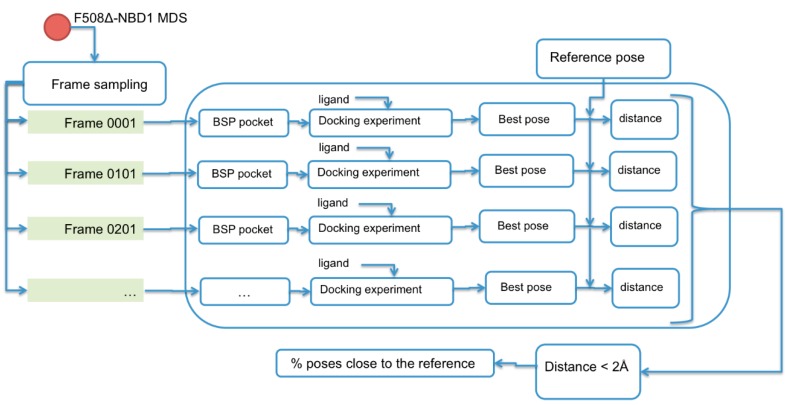
Workflow of the applied ligand-protein inverse docking technique.

In order to try to explain the experimental RU values obtained by SPR analysis in relation to the pocket availability at equilibrium of the ΔF508-NBD1, we used a ligand-protein inverse docking technique to obtain a measure of how many ΔF508-NBD1 structural conformations along the MDS are able to bound to their respective ligands (Figure 11).

We implemented a protein pocket dataset that includes the three pockets (BSP1, BSP2, BSP3) obtained from the workflow illustrated in Figure 3, and performed docking analysis of each pocket against a selection of 1000 time-equidistant frames along the MDS of the ΔF508-NBD1 (one each 200 ps). The computations were carried out using local docking with Auto-Dock Vina. For each ligand/ΔF508-NBD1 docking simulation, the lowest energy complex was identified and for each ligand, all docked poses over the 1000 frames were grouped by similar ligand-pocket distance between the docking pose and the reference pose, the latter obtained considering the most frequent cluster of the dynamic of the complex. The results were then compared to the mean value of the binding energy of the group, allowing the evaluation of the number of ΔF508-NBD1 conformations able to bind to each compound (Table S2).

#### 4.1.6. Trajectory Analysis

MDS trajectories were analysed using the root mean square deviation (RMSD) and the root mean square fluctuation (RMSF) techniques plus the hydrophobic surface (HS). RMSD was used as a measure of the stability of the unbound proteins compared to the crystallographic structures and as a measure of the ligand complex along the dynamics compared to its starting pose obtained from docking. RMSF was used to determine the fluctuating regions in ΔF508-NBD1, highlighting the differences in flexibility in respect to the WT form of the protein.

HS area was computed with the per-residue Shrake-Rupley method [46] using 1200 probes with a 1.4 Å radius to evaluate the ΔF508-NBD1 hydrophobic exposure area during the simulations.

### 4.2. Reagents and Recombinant Proteins

Murine WT NBD1 and ΔF508-NBD1 were obtained from CFTR Folding Consortium [37]. K8 was purchased by Pro-Spec-Tany TechnoGene Ltd. (Ness Ziona, Israel). The reagents 1-ethyl-3-(3-diaminopropyl)carbodiimide hydrochloride (EDC) and *N*-hydroxysuccinimide (NHS) were purchased from GE Healthcare (Milwaukee, WI, USA).

### 4.3. Surface Plasmon Resonance (SPR) Binding Assay

SPR measurements were performed on a BIAcore X-100 instrument (GE-Healthcare, Milwaukee, WI, USA), using research-grade CM5 carboxyl-methyl-dextran–coated sensor chips (GE-Healthcare). The murine ΔF508-NBD1 was resuspended at 20 µg/mL in 10 mM sodium acetate pH 4.0 and allowed to react with the first flow cell of a CM5 sensor chip pre-activated with 50 μL of 0.2 M EDC and 0.05 M NHS and deactivated by injection of HCl–ethanolamine. This procedure led to the immobilization of 11,000 RU, equal to approximately 400 fmol/mm^2^ of protein. Similar results were obtained for the injection of BSA (20 µg/mL) onto the second flow cell of each sensor chip, used as a negative control and for blank subtraction. The appropriate immobilization of ΔF508-NBD1 was checked by injecting increasing concentrations of K8 in PBS, 0.05% surfactant P20 and 5% DMSO, pH 7.4 over the sensor chip for 90 s at 10 μL/min, 25 °C and then washed until dissociation was observed. The inclusion of 5% DMSO in the running buffer (not necessary for protein-protein interaction analysis) and the short association time were chosen to validate ΔF508-NBD1 binding availability in the experimental conditions used for the subsequent analysis of the compounds (see below).

#### 4.3.1. Direct Binding Analysis

For the evaluation of their capacity to directly bind to ΔF508-NBD1, increasing concentrations of the various compounds of the data-set were resuspended in PBS, 0.05% surfactant P20 and 5% DMSO, pH 7.4, injected over ΔF508-NBD1 or BSA surfaces for 90 s at 10 μL/min, 25 °C and then washed until dissociation was observed. Steady state analysis were performed by fitting the proper form of Scatchard’s equation for the plot of the bound RU at equilibrium versus the compound concentration in solution. When saturation binding was reached, *K_d_* values were calculated. In the case no saturation could be reached due the limited solubility of the compound under test, the software provided a *K_d_* value expected on the bases of the curve’s trend.

In a further series of experiments, a saturating amount of K8 (1 µM) was injected on the ΔF508-NBD1 surface and allowed to reach an equilibrium binding. Then, the various compounds of the data-set (150 µM) were injected and their capacity to affect the binding of K8 to ΔF508-NBD1 evaluated.

#### 4.3.2. Competition Assays

K8 was used since, as a representative ΔF508-CFTR chaperones, has been repeatedly used for SPR studies [4,34,47] and its binding site in ΔF508-CFTR has been mapped [34]. To perform competition assays for the binding to ΔF508-NBD1 between the various compounds and K8, the latter was resuspended at 20 µg/mL in 10 mM sodium acetate pH 4.0 and allowed to react with the first flow cell of a CM5 sensor chip pre-activated with 50 μL of 0.2 M EDC and 0.05 M NHS and deactivated by injection of HCl–ethanolamine. This procedure led to the immobilization of 7800 RU of the protein, (equal to approximately 146 fmol/mm^2^). Similar results were obtained for the injection of BSA (10 μg/mL) onto the second flow cell of each sensor chip, that was then used as a negative control and for blank subtraction. ΔF508-NBD1 (at 600 nM) in the absence or in the presence of the various compounds (all at 500 μM) in 0.05% surfactant P20 and 5% DMSO, pH 7.4, was injected over the K8 or BSA surfaces for 90 s at 10 μL/min, 25 °C and then washed until dissociation was observed.

## Figures and Tables

**Table 1:** Affinity values (*K_d_*) of the interactions of CF putative drugs with ΔF508-NBD1 calculated by SPR analysis. *K_d_* values were derived by Scatchard’s plot analysis of the equilibrium binding data. The results shown are the mean ± standard deviation (S.D.) of two-three separate analyses. n.d.: not determinable.

Interaction	*K_d_* (μM)
VX770/ΔF508-NBD1	n.d.
C4a/ΔF508-NBD1	n.d.
VX809/ΔF508-NBD1	24.2 ± 6.2
4/ΔF508-NBD1	99.3 ± 14.5
5/ΔF508-NBD1	40.3 ± 2.5
6/ΔF508-NBD1	197.9 ± 4.5
7/ΔF508-NBD1	n.d.

## Supplementary Materials

Supplementary materials are available online.
