# Supplementary material for: Speeding Up the Identification of Cystic Fibrosis Transmembrane Conductance Regulator-Targeted Drugs: An Approach Based on Bioinformatics Strategies and Surface Plasmon Resonance
Source: Molecules. 2018 Jan 8;23(1):120. doi: 10.3390/molecules23010120 (PMC6017603; doi:10.3390/molecules23010120)
Supplement: Supplementary file 1 [file molecules-23-00120-s001.pdf]

molecules

SUPPLEMENTARY FILES

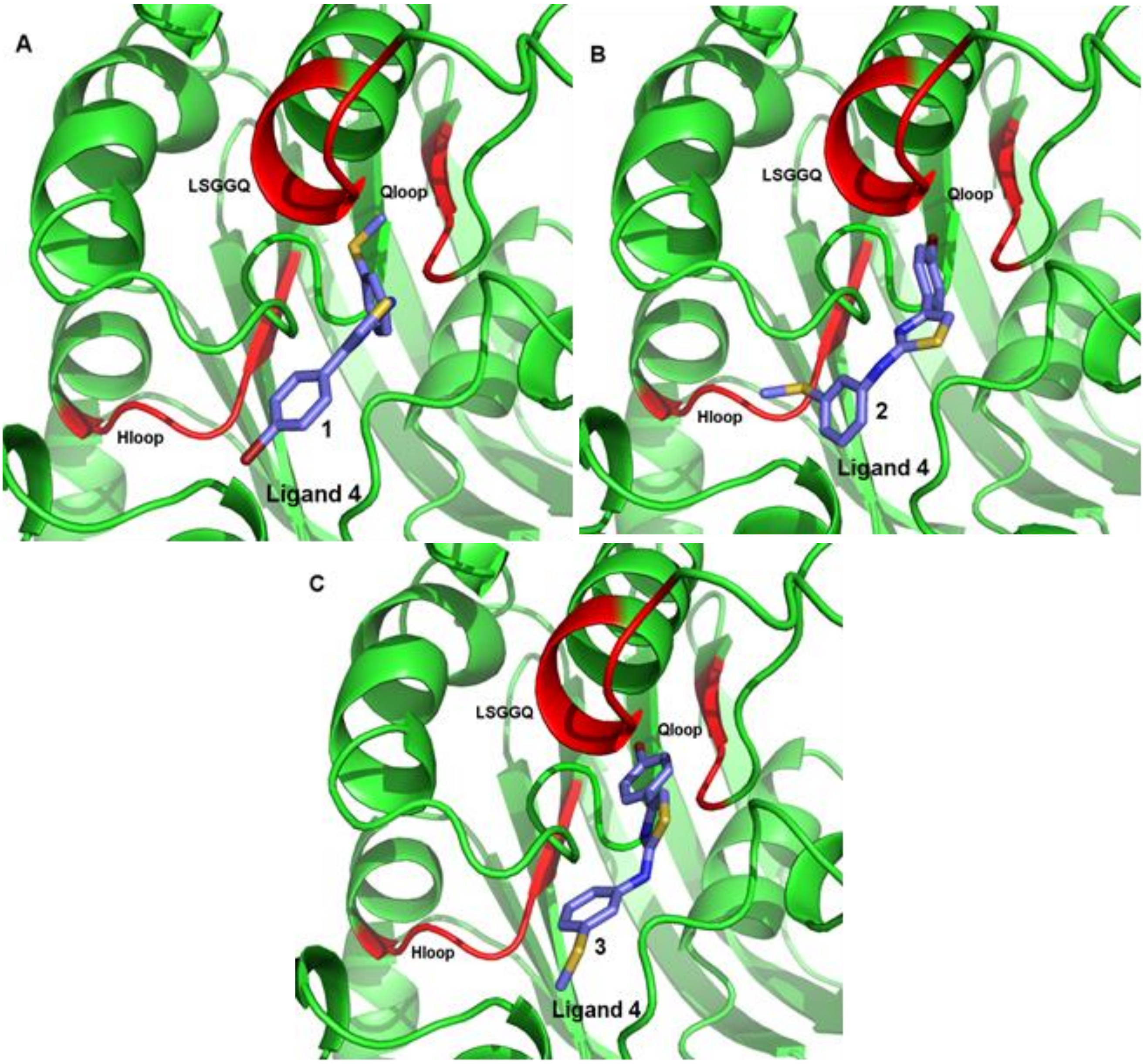

Figure S1. Inverse orientations of the AAT derivative **4** from docking simulations. The ligand is represented as sticks and the protein as ribbon. The different orientations are indicated with Arabic numbers.

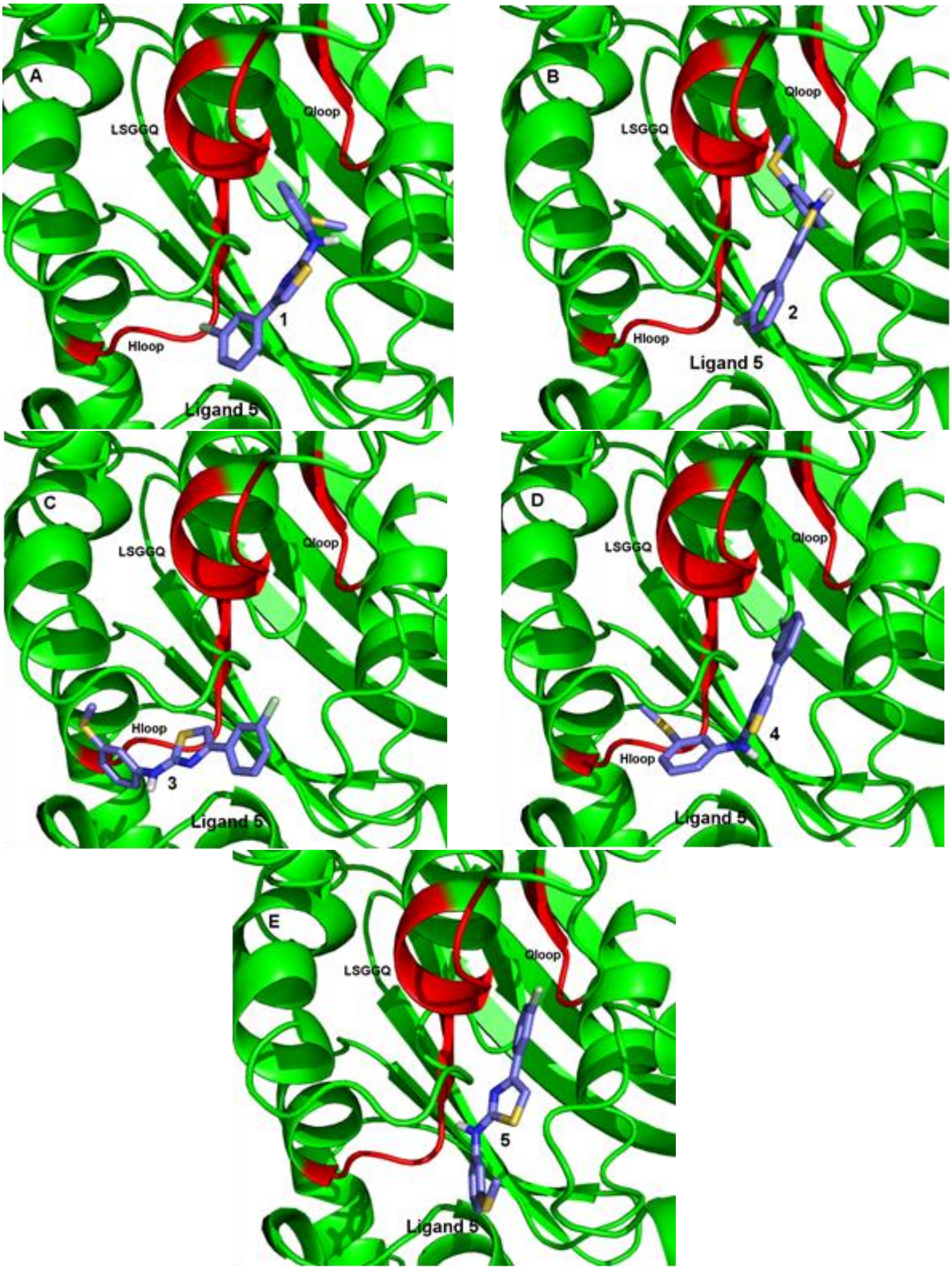

Figure S2: Inverse orientations of the AAT derivative **5** from docking simulations. The ligand is represented as sticks and the protein as ribbon. The different orientations are indicated with Arabic numbers.

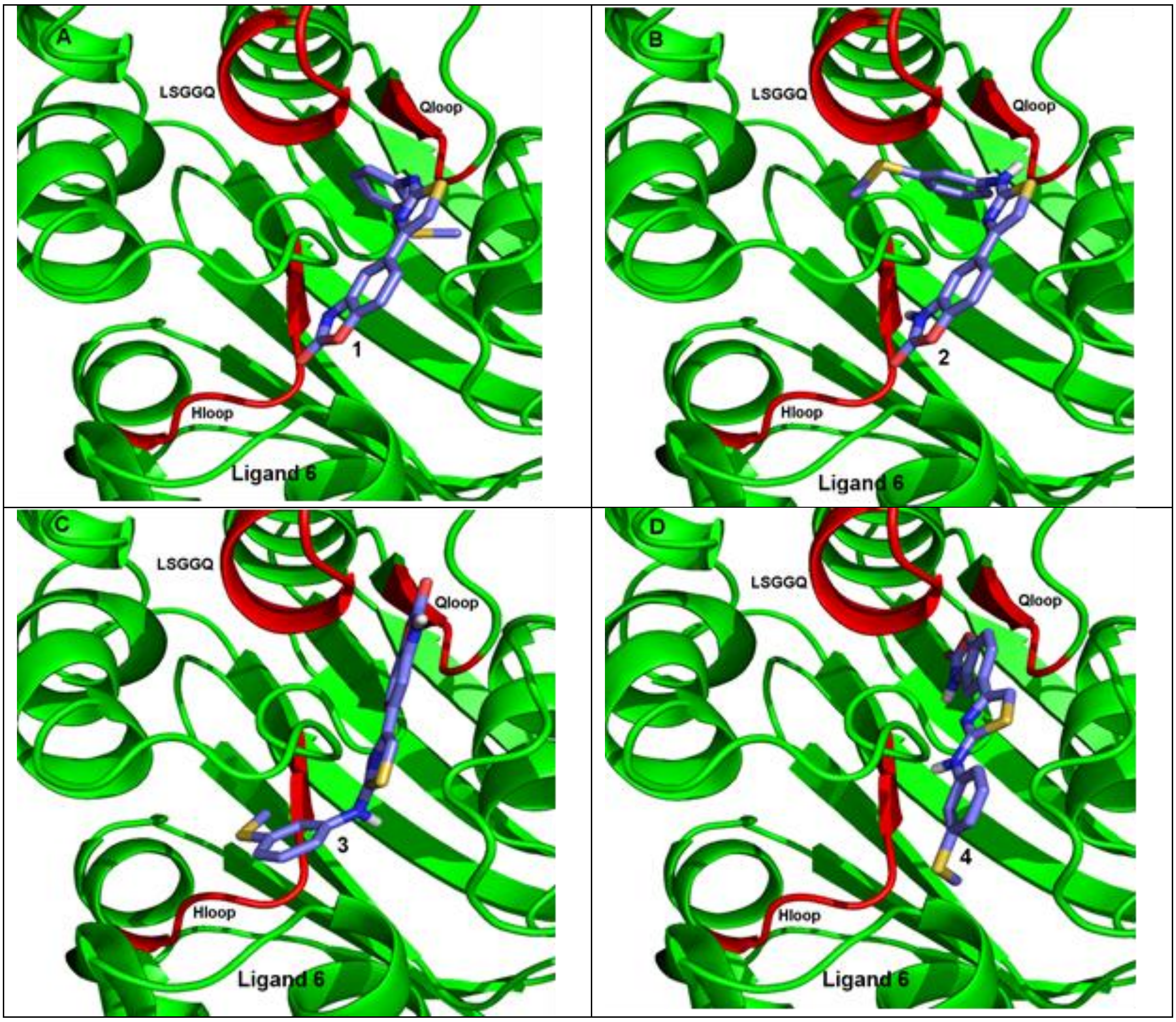

Figure S3: Inverse orientations of the AAT derivative **6** from docking simulations. The ligand is represented as sticks and the protein as ribbon. The different orientations are indicated with Arabic numbers.

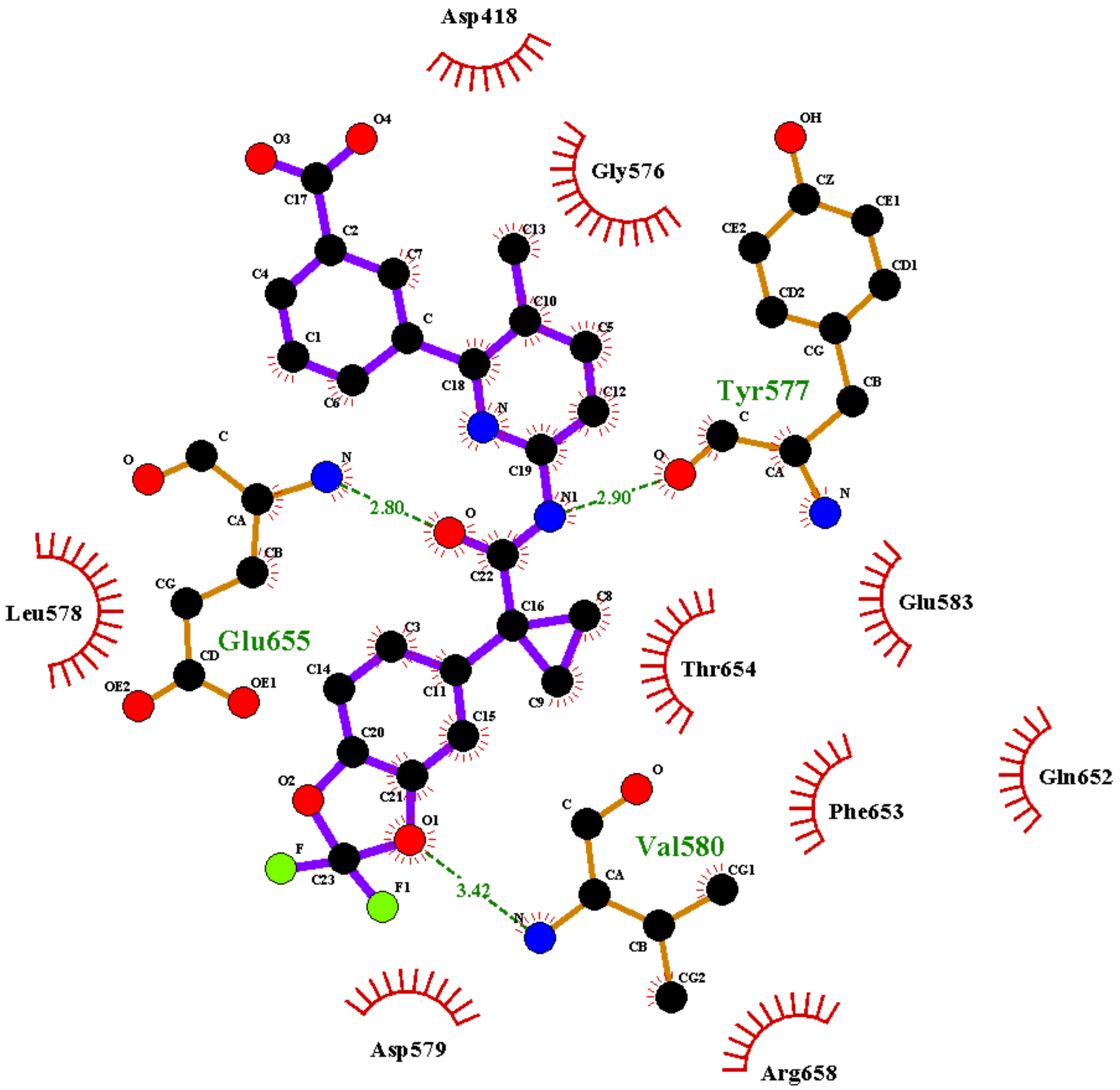

Figure S4: Ligplot interaction map of compound VX809.

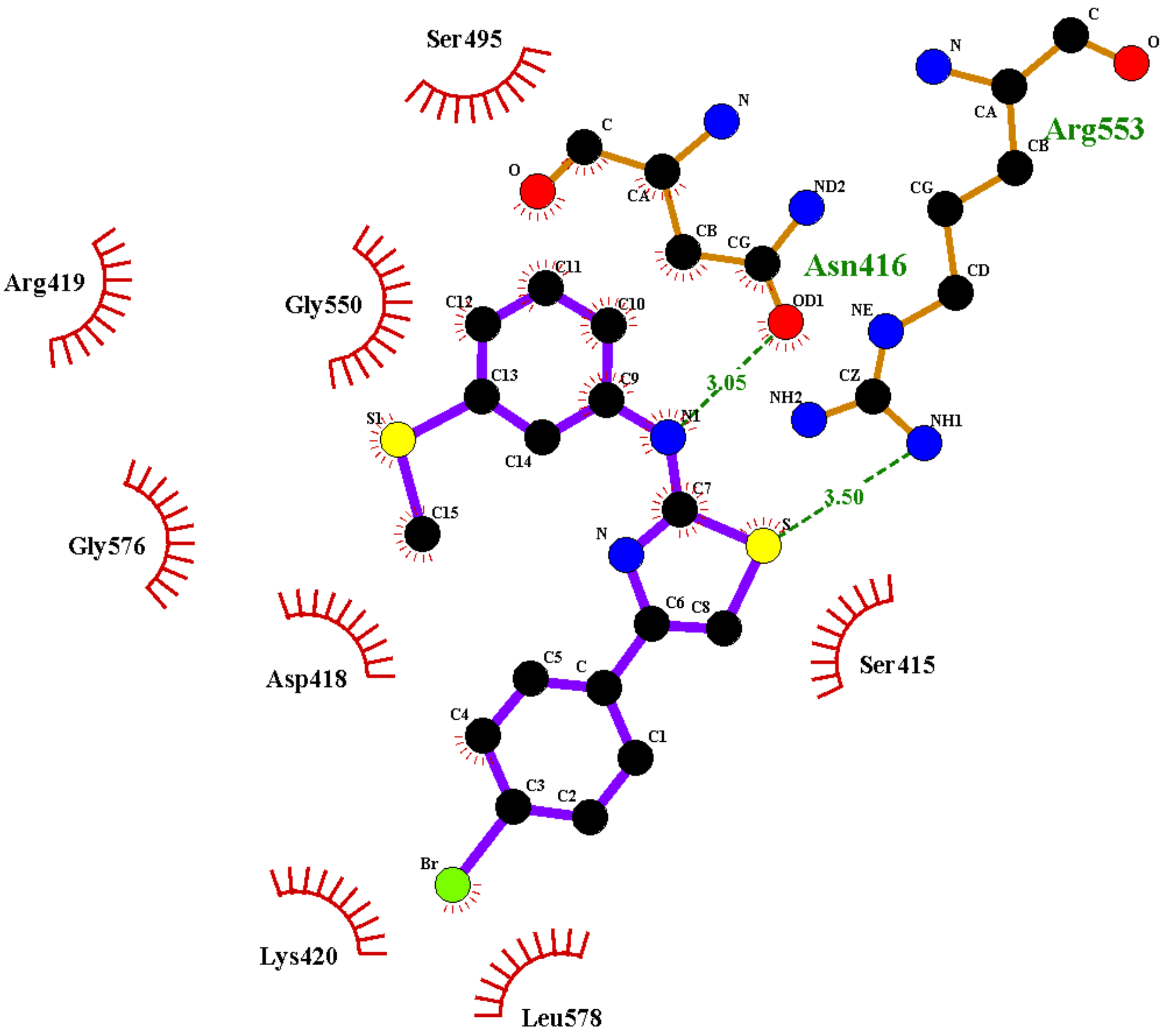

Figure S5: Ligplot interaction map of the AAT derivative **4**.

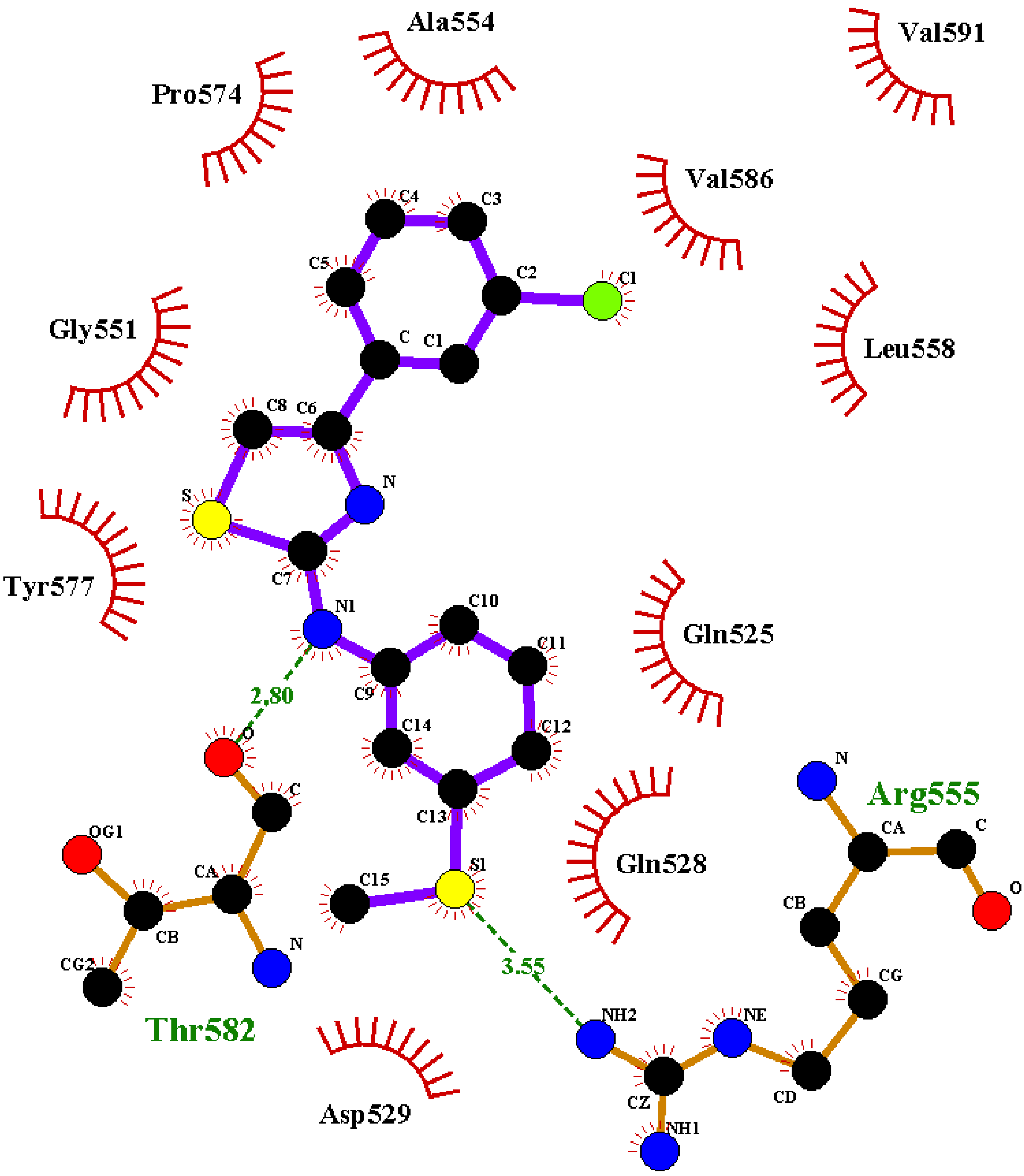

Figure S6: Ligplot interaction map of the AAT derivative **5**.

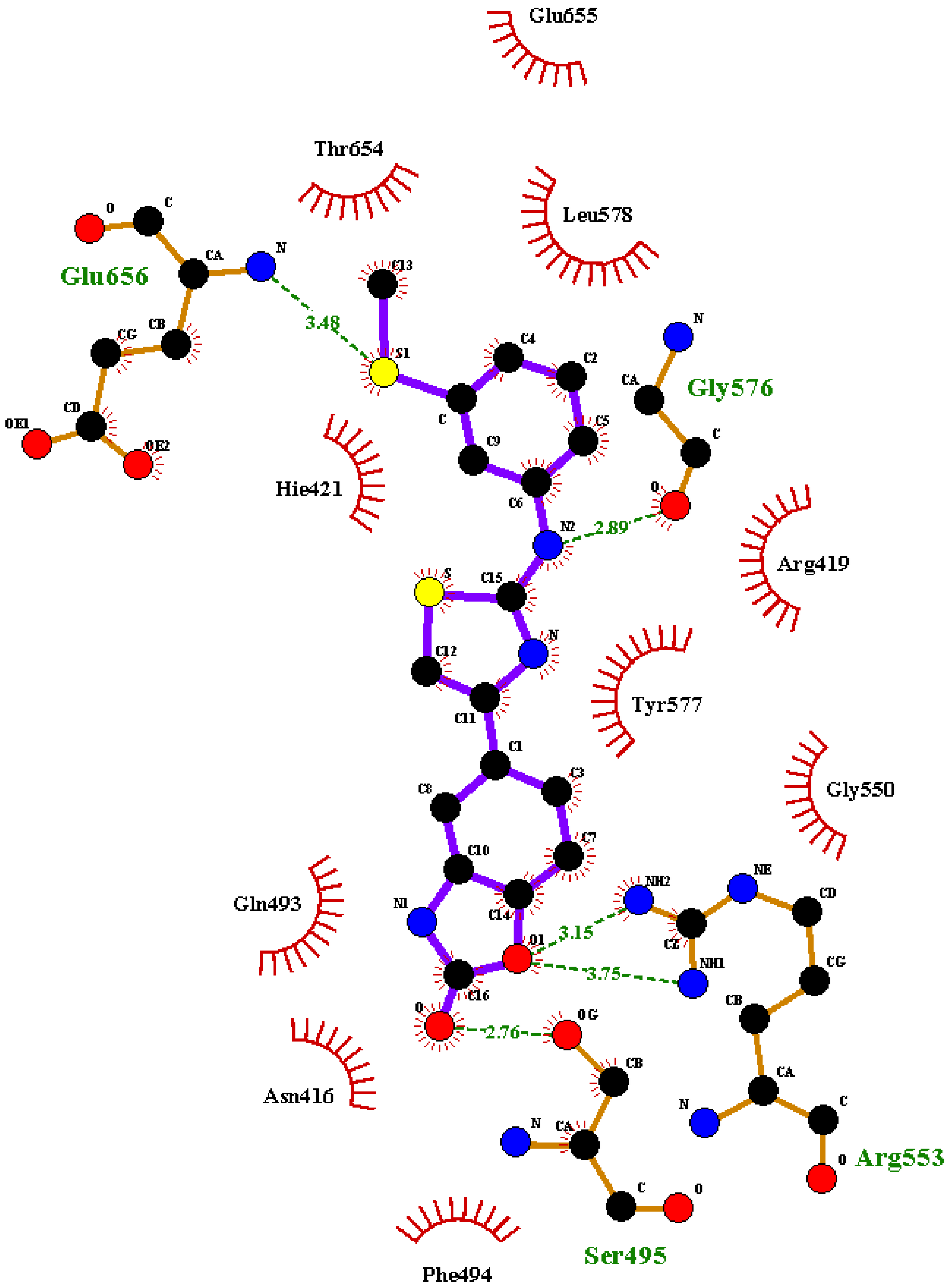

Figure S7: Ligplot interaction map of the AAT derivative **6**.

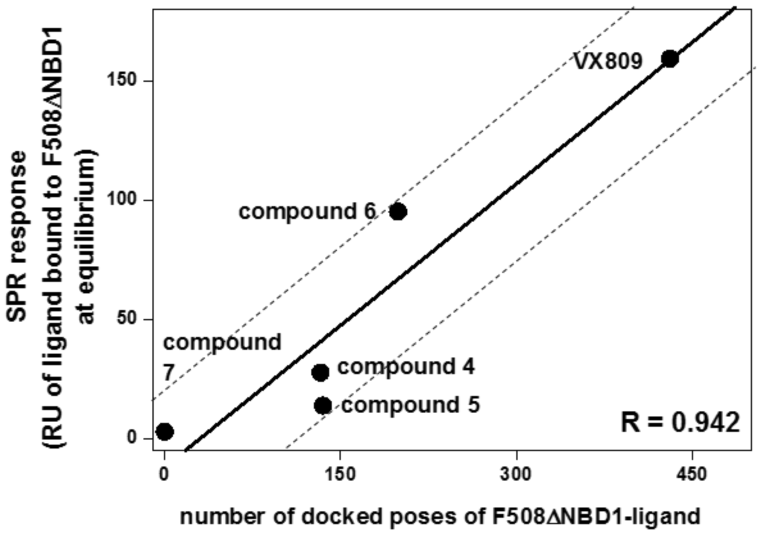

Figure S8: Plot of the correlation existing between the number of docked poses and SPR-generated values of maximal RU bound at equilibrium for the interaction of immobilized-ΔF508-NBD1 with the various ligands. SPR responses taken from a single experiment out of three giving consistent results.

Table S1. AAT derivatives clustered by structural conformation (RMSD) and grouped by similar energy of the docking.

AAT derivatives	Cluster	Lowest binding energy (kcal/mol)	Number of conformations in the cluster
	1	−7.66	14
4	2	−7.53	41
	3	−7.46	17
	1	−8.9	14
	2	−8.85	11
5	3	−8.84	7
	4	−8.58	27
	5	−8.24	22
	1	−10.05	65
6	2	−9.37	2
	3	−9.30	6
	4	−9.12	21

Table S2. Relationship existing between docking poses along the ΔF508-NBD1 trajectory and SPR-generated values of maximal RU bound at equilibrium. The number of docking poses obtained using 1000 conformations of ΔF508-NBD1 MD and RU units from SPR analysis at equilibrium are reported for each ligand. The RU units from SPR analysis are the mean ± s.d. of two-three separate analyses.

Ligand	Docking poses of ΔF508-NBD1-ligand	RU of ligand bound to ΔF508-NBD1 at equilibrium
**VX809**	431 (48%)	163.7 ± 23.7
**4**	133 (15%)	32.0 ± 4.2
**5**	135 (15%)	13.0 ± 1.4
**6**	199 (22%)	114.0 ± 5.6
**7**	n.d. *	n.d. **

* Compound **7** was instable from the first MD runs. ** The binding of compound **7** to ΔF508-NBD1 is not dose-dependent and varies from 0 to 4 RU among the doses tested. n.d.: not determinable.
